# Metropolitan Asylums Board Report

**Published:** 1923-09

**Authors:** 


### METROPOLITAN ASYLUMS BOARD.

### ITS VARIED ACTIVITIES.

THE annual report of the Metropolitan Asylums Board for the year 1922-23, which has just been issued, teems with information about the interesting work of this inadequately-named Board. Who would suppose, unless he dipped into this volume, that the duties of the Board include the provision and management of hospitals and other institutions for infectious diseases, for tuberculosis and for children suffering from specified contagious diseases and those requiring special treatment in general hospitals or convalescent homes, in addition to institutions for mental defectives and epileptics, boys for training for sea service, and for the casual poor, and that they provide ambulance and transport services ?

### Fevers.

The Board point out that the year 1922 was the third from the commencement of the recent high prevalence of infectious disease in the Metropolis, which, though it reached its culminating point in the year 1921, actually started in 1920 :—

The incidence of scarlet fever and diphtheria in London fluctuates greatly from year to year ; as a rule three or four high years are followed by three or four low ones, and since 1903 very regular seven-yearly extensions have occurred. The years 1907, 1914 and 1921 form crests of the waves and each of these years is preceded and followed (with one exception) by a year in which the incidence, though not so high as that of the crest-year, is still at a very high level. The one exception has been the past year 1922, when according to anticipation the incidence of disease, while substantially lower than that of 1921, should have been still very high. Expectations, however, were not realised ; on the contrary, the total incidence of disease fell with remarkable rapidity towards the normal.

### Tuberculosis.

The Board's institutions for adult cases suffering from tuberculosis include two sanatoria for early cases, three hospitals for advanced cases and one hospital for surgical tuberculosis. For children suffering from tuberculosis three institutions have been provided—two at the seaside and one in the country. Additional accommodation for children is found in one of the Board's general hospitals for children. The Board have some interesting observations about sanatorium life :—

Sanatorium life, while strict in the essential details of treatment, has to be made reasonably attractive by the elimination of unnecessary discomforts and restrictions, and by the provision of suitable recreation. The patients have to live under open-air conditions, have to be warmly clad and to have a full diet. The main principle of sanatorium treatment is to reduce activity of the disease and associated symptoms by rest, and when control of the tuberculous poisoning is thus secured, to build up physical capacity and resistance to the disease by careful graduation of exercise, first by walking, until the patient can walk without harm five or six miles a day, and then by manual work. The latter is obtained either by graded gardening work or by such handicrafts as carpentry, leather work and basketry.

### Eliminating the Poor-Law Atmosphere.

The Board feel that they have special responsibilities towards the sick children who come under their care. They have consistently recognised an obligation to do more than merely ameliorate the temporary disability for the treatment of which the children are primarily entrusted to its charge. Every effort is made so to mould them that their outlook on life shall in many cases be completely changed and their character so formed as to convert them into useful citizens. To this end the report says :—

The Poor-law atmosphere has been practically eliminated from all the children's homes and hospitals, each of which is individualised and has lost what is implied in the term " an institutional character." Education is carried on wherever possible, bedside teaching being employed where necessary ; excursions to places of interest and of educational value are arranged ; the children are encouraged to organise their own entertainments ; cinematograph films are shown and prove to be both instructive and amusing to children who may never have seen one before.

### Sunlight for Sick Children.

The Board's General Hospital for Children at Carshalton (Queen Mary's Hospital) contains 850 beds, the accommodation being provided in sixteen ward blocks and in two isolation buildings. Special attention is given to treatment by sunlight and fresh air :—

In 1912, verandahs, each containing thirty beds, were added to ten of the ward blocks, thus enabling 300 children to lie in the open air, winter and summer, day and night, almost completely free from colds and from infectious diseases. Competition for these verandah beds is very keen in the summer months, and even in the winter the children get accustomed to the open air and thoroughly enjoy it. Extensive use is made of direct sunlight, and the courtyards, open only to the south, with the verandahs on the three remaining sides, are ideal for the carrying out of this treatment. The orthopædic carriages designed by the medical superintendent have proved of value, particularly in carrying out sunlight treatment, for they enable the patients who are accommodated indoors to reap to the full during the day the benefits derived from light, air and sunshine.

### Lifting up the Casual Ward.

The period under review is particularly noteworthy for a very important step in dealing with the casual poor which has been taken by the Board :—

A scheme has been adopted for the reservation of one of the wards as a hostel for men to which cases likely to respond to help will be sent from the casual wards and the Night Office. At this hostel, in which cases will be allowed to remain for an extended period, the work will consist solely in endeavouring, with the co-operation of the charitable agencies and the employment exchanges, to re-establish the men in employment. The Ministry of Health have now sanctioned, for an experimental period of one year, the institution of the scheme, with the special regulations and increased dietary which are essential to its success, and it is hoped that the hostel, for which it has been decided to use the Holborn Ward, will be opened shortly.

### Industrial Training.

The industrial work carried out at Darenth Training Colony for improvable imbeciles, mental defectives, and the feeble-minded has, since the transfer to the colony of the patients from the Bridge Training Home, expanded considerably and has now reached a large volume. The activities of the Colony comprise bookbinding, printing and upholstery shops and many other forms of industrial effort.

